# Clinical prediction scores and early anticoagulation therapy for new-onset atrial fibrillation in critical illness: a post-hoc analysis

**DOI:** 10.1186/s12872-021-02235-8

**Published:** 2021-09-08

**Authors:** Masaaki Sakuraya, Takuo Yoshida, Yusuke Sasabuchi, Shodai Yoshihiro, Shigehiko Uchino

**Affiliations:** 1 Department of Emergency and Intensive Care Medicine, JA Hiroshima General Hospital, Jigozen 1-3-3, Hatsukaichi, Hiroshima 738-8503 Japan; 2 Intensive Care Unit, Department of Anesthesiology, Jikei University School of Medicine, Tokyo, Japan; 3 Present Address: Department of Intensive Care Medicine, Tokyo Women’s Medical University, Tokyo, Japan; 4 Data Science Center, Jichi Medical University, Shimotsuke, Tochigi Japan; 5 Pharmaceutical Department, JA Hiroshima General Hospital, Hatsukaichi, Hiroshima Japan; 6 Department of Anesthesiology and Critical Care Medicine, Jichi Medical University Saitama Medical Centre, Saitama, Japan

**Keywords:** Anticoagulation therapy, Critical illness, Ischemic stroke, New-onset atrial fibrillation, Rhythm control therapy

## Abstract

**Purpose:**

This study sought to describe the epidemiology of anticoagulation therapy for critically ill patients with new-onset atrial fibrillation (NOAF) according to CHA2DS2-VASc and HAS-BLED scores and to assess the efficacy of early anticoagulation therapy.

**Method:**

Adult patients who developed NOAF during intensive care unit stay were included. We compared the patients who were treated with and without anticoagulation therapy within 48 h from AF onset. The primary outcome was a composite outcome that included mortality and ischemic stroke during the period until hospital discharge.

**Results:**

In total, 308 patients were included in this analysis. Anticoagulants were administered to 95 and 33 patients within 48 h and after 48 h from NOAF onset, respectively. After grouping the patients into four according to their CHA2DS2-VASc and HAS-BLED bleeding scores, we found that the proportion of anticoagulation therapy administered was similar among all groups. After adjustment using a multivariable Cox regression model, we noted that early anticoagulation therapy did not decrease the composite outcome (adjusted hazard ratio [HR] 0.77; 95% confidence interval [CI] 0.47‒1.23). However, in patients without rhythm control drugs, early anticoagulation was significantly associated with better outcomes (adjusted HR 0.46; 95% CI; 0.22‒0.87, *P* = 0.041).

**Conclusions:**

We found that clinical prediction scores were supposedly not used in the decision to implement anticoagulation therapy and that early anticoagulation therapy did not improve clinical outcomes in critically ill patients with NOAF.

*Trial registration* UMIN-CTR UMIN000026401. Registered 5 March 2017.

**Supplementary Information:**

The online version contains supplementary material available at 10.1186/s12872-021-02235-8.

## Introduction

Atrial fibrillation (AF) is one of the most common arrhythmias in the intensive care unit (ICU) [1–3]. It is associated with increased length of hospital stay, in-hospital mortality, ischemic stroke, and heart failure [4–10]. Clinical guidelines in the general population recommend anticoagulation therapy to prevent stroke in moderate or high-risk patients, as defined by clinical scores, including CHADS2 and CHA2DS2-VASc scores [11–14]. Bleeding risk is also important for prescribing anticoagulants. The HAS-BLED bleeding risk score has been reported to be useful for predicting major bleeding, cardiovascular events, and mortality in the general population [15–17].

Previous studies have reported that anticoagulants were prescribed for less than 40% of critically ill patients with new-onset AF (NOAF) [18–21], although most critically ill patients were considered to be at high risk of ischemic stroke (CHA2DS2-VASc score ≥ 2) [22]. Although critically ill patients may have increased bleeding risks, only a few studies have evaluated clinical scores, including the HAS-BLED score, for predicting bleeding risk in critically ill patients with AF. Anticoagulation therapy in critically ill patients with AF may not be common because of the uncertainty in evaluating bleeding risks in these patients.

The widely used threshold of AF duration for prescribing anticoagulants is “at least 48 h” in a general setting [14]. An observational study in a general population reported that early anticoagulation therapy within 48 h from AF onset decreased thromboembolic complications in patients with a high risk of ischemic stroke (CHA2DS2-VASc score ≥ 2) [23]. NOAF in critically ill patients does not usually last longer than 48 h [24]. Considering the high risk of ischemic stroke in critically ill patients, earlier anticoagulation therapy may contribute to a better outcome in critically ill patients with NOAF, similar to high-risk patients in general settings. Previous studies have found that anticoagulation therapy for critically ill patients with AF did not prevent in-hospital ischemic stroke [20, 21, 25]. However, the timing of the anticoagulation therapy after AF onset has not been assessed in previous studies in the ICU.

We conducted a post-hoc analysis of the Atrial Fibrillation Treatment Evaluation Registry in the ICU study (AFTER-ICU), which was a prospective multicenter cohort study of NOAF in the general ICU population. The aim of this analysis was to describe the epidemiology of anticoagulation therapy for critically ill patients with NOAF, according to the CHA2DS2-VASc and HAS-BLED scores, and to assess the efficacy of early anticoagulation in these patients. We hypothesized that early anticoagulation therapy (within 48 h from AF onset) could improve neurological outcomes in critically ill patients with NOAF.

## Methods

### Study design and population

This study was a post-hoc analysis of the AFTER-ICU study conducted in 32 ICUs in Japan from April 1, 2017 to March 31, 2018. The participants included in the AFTER-ICU study were adult patients who developed NOAF during ICU stay. The exclusion criteria of the original study were as follows: (i) age under 18 years, (ii) an AF history, (iii) discharge from the ICU within 24 h of ICU admission, (iv) admission to the ICU after cardiac surgery or cardiac arrest, (v) having a pacemaker at AF onset, (vi) withholding or withdrawal from medical therapy at AF onset, and (vii) refusal to participate in this study [24]. Additionally, patients were excluded from this post-hoc analysis if they died or were discharged from the ICU within 48 h after AF onset, had stroke or bleeding complications within 2 days after AF onset, or were administered any anticoagulants at AF onset.

### Variables and measurements

Baseline information, including age, severity of illness (Acute Physiology and Chronic Health Evaluation II [APACHE II] score [26] and Sequential Organ Failure Assessment [SOFA] score [27]), presence of sepsis, requirement for mechanical ventilation and renal replacement therapy (RRT), and length of ICU stay, were collected. We also recorded the following information during a period of 7 days after the initial AF onset or throughout the ICU stay, whichever was shorter: antiarrhythmic agent use, anticoagulant use, direct-current cardioversion, and date and time of restoration of sinus rhythm (SR). Restoration of SR was defined as SR sustained for longer than 24 h after the conversion from AF to SR [28]. AF duration was calculated as the time from the initial AF onset to the first restoration of SR. CHA2DS2-VASc score and HAS-BLED bleeding risk score [15] were calculated from the data of the original study.

### Exposure and outcomes

We compared NOAF patients who were treated with any anticoagulants within 48 h from AF onset (the Early group) and those without anticoagulation therapy within this period (the Non-early group). Anticoagulation therapy was defined as the use of the following anticoagulants during a 7-day period after the initial AF onset or throughout the ICU stay, whichever was shorter: subcutaneous heparin injection, continuous heparin intravenous injection, warfarin, and direct oral anticoagulant.

Patients were followed up until hospital discharge. We compared a composite outcome, which included mortality and ischemic stroke from AF onset to hospital discharge. Information on bleeding events, based on the Bleeding Academic Research Consortium (BARC) definition for bleeding, during ICU stay was also collected (Additional file 1: Table S1) [29].

### Statistical analysis

Data are expressed as medians with interquartile ranges or numbers with corresponding percentages, as appropriate. In all analyses, the number of cases with missing data was reported, and such cases were excluded from each analysis. Baseline characteristics were compared between the Non-early group and the Early group. Continuous variables were compared using Student’s *t*-test or the Mann‒Whitney U test, according to the data distribution. Dichotomous variables were analyzed using the chi-square test or Fisher’s exact test. Time-to-event data were described using the Kaplan–Meier plot, and the log-rank test was used to compare the two groups. We censored an observation if a patient was surviving at hospital discharge. Cox proportional hazard models were used to estimate the effect of early anticoagulation therapy on hospital mortality or ischemic stroke after AF onset. Co-variables known to be associated with mortality in critically ill adults (i.e., age, sex, APACHE II score, CHA2DS2-VASc score, infection, use of mechanical ventilation, and RRT) were identified a priori and subsequently encoded into the models [26, 30–33]. The HAS-BLED bleeding risk score was also entered into these models because it was reported to be associated with mortality in non-critically ill patients with AF [16, 17]. We included interaction terms between the early anticoagulation therapy and rhythm control drugs, rate control drugs, and cardioversion within 48 h from AF onset to evaluate the effect modification. Then, the effect of early anticoagulation therapy was evaluated in these subgroups. All statistical tests were two-sided, and a *p* value < 0.05 was defined as indicating statistical significance. All data were analyzed using JMP 12.2.0 (SAS Institute Inc., Cary, NC, USA).

## Results

**Fig. 1:**
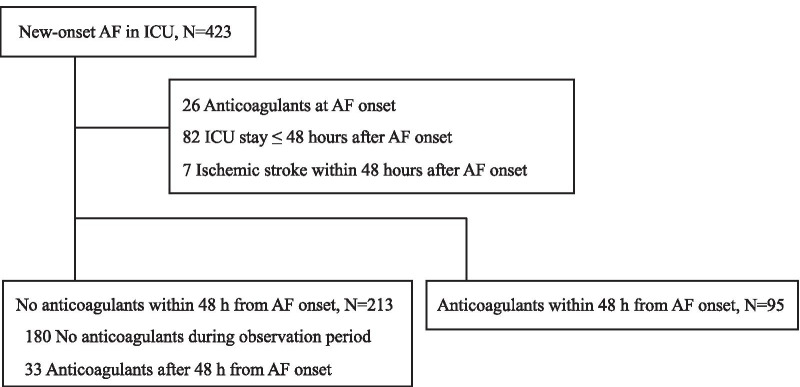
Patient flow diagram. AF, atrial fibrillation; ICU, intensive care unit

In total, 14,348 adult non-cardiac surgical patients were admitted to the ICU during the study period. Of these, 423 patients (2.9%) had NOAF, and after applying the inclusion and exclusion criteria, 308 patients were included for this analysis (Fig. 1). Anticoagulants were administered in 95 patients within 48 h from AF onset and in 33 patients more than 48 h after onset. Anticoagulants were never used for 180 patients during the observation period. No patients were lost to follow-up until hospital discharge.

**Fig. 2:**
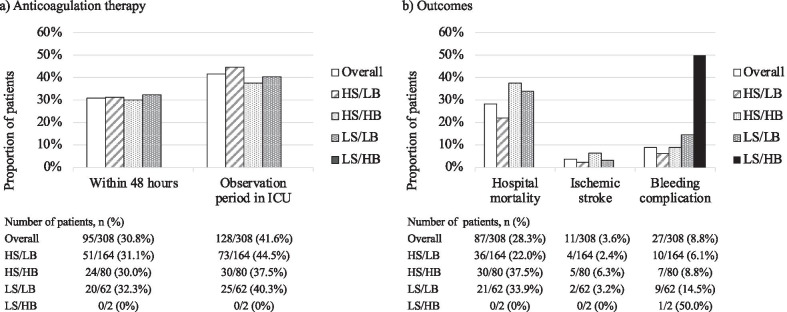
Anticoagulation therapy and clinical outcomes in the group divided by CHA2DS2-VASc and HAS-BLED scores. **a)** For anticoagulation therapy and **b)** for mortality and the incidence of ischemic stroke and bleeding complocations. Stroke risk was determined using the CHA2DS2-VASc score (high risk of stroke [HS] ≥ 2; low risk of stroke [LS] < 2), and bleeding risk was determined using the HAS-BLED score (high risk of bleeding [HB] ≥ 3; low risk of bleeding [LB] < 3). We divided the patients into four groups based on both risks (HS/LB, HS/HB, LS/LB, and LS/HB). Only two patients were included in the LS/HB group

After dividing the patients into four groups based on CHA2DS2-VASc and HAS-BLED bleeding scores, we found that the proportion of patients treated with anticoagulation therapy was similar among all groups, except for the low risk of stroke and the high risk of bleeding group, which included only two patients (Fig. 2). In addition, 164 patients (53.3%) were considered to be at high risk of stroke (CHA2DS2-VASc score ≥ 2) and at low risk of bleeding (HAS-BLED bleeding score < 3), only 31.1% and 44.5% of whom received anticoagulation therapy within 48 h from AF onset and during the whole observation period, respectively. Details of the CHA2DS2-VASc score and HAS-BLED bleeding risk score are shown in Additional file 1: Tables S2 and S3.

Patient demographics and clinical characteristics are shown in Table 1, and the physiological data and laboratory test results are shown in Additional file 1: Table S4. Of the 308 patients, 208 (67.5%) were male, and most patients (194 patients, 63.0%) were medical patients. A total of 212 patients (68.8%) received mechanical ventilation, and 83 patients (27.0%) received RRT at AF onset. Most patients had a CHA2DS2-VASc score ≥ 2 (244 patients, 79.2%), and 82 patients (26.6%) had a HAS-BLED bleeding risk score ≥ 3. Neither score was significantly different between the Early and the Non-early group. Disease severity scores (APACHE II and SOFA scores) for the Early group were lower than those for the Non-early group. Hemodynamic data and the proportion of patients using vasoactive drugs were similar between the two groups. The Early group had a higher platelet count and lower bilirubin, creatinine, and lactate levels than the Non-early group.

**Table. 1:** Patient characteristics: non-early group vs. early group

	OverallN = 308	Non-early groupN = 213	Early groupN = 95	*P* value
Age, years	75 (66–82)	74 (66–81)	75 (66–83)	0.495
Male, n (%)	208 (67.5%)	144 (67.6%)	64 (67.4%)	0.967
Body mass index, kg/m^2^	22.7 (19.6–25.3)	22.5 (19.4–25.3)	22.9 (20.1–25.2)	0.824
Patient category				0.699
Non-scheduled surgery, n (%)	78 (25.3%)	51 (23.9%)	27 (28.4%)	
Scheduled surgery, n (%)	36 (11.7%)	25 (11.7%)	11 (11.6%)	
Medical, n (%)	194 (63.0%)	137 (64.3%)	57 (60.0%)	
APACHE II score at ICU admission	24 (19–30)	25 (20–30)	23 (18–27)	0.039
SOFA score at AF onset	8 (6–11)	8 (6–12)	7 (5–9)	0.009
Chronic hemodialysis, n (%)	17 (5.5%)	14 (6.6%)	3 (3.2%)	0.226
CHA2DS2-VASc score	3 (2–4)	3 (2–4)	3 (2–4)	0.859
CHA2DS2-VASc ≥ 2, n (%)	244 (79.2%)	169 (79.3%)	75 (79.0%)	0.937
HAS-BLED bleeding risk score	2 (1–3)	2 (1–3)	2 (1–3)	0.562
HAS-BLED ≥ 3, n (%)	82 (26.6%)	58 (27.2%)	24 (25.3%)	0.718
Infection at AF onset, n (%)	230 (74.7%)	157 (73.7%)	73 (76.8%)	0.559
From ICU admission to AF onset, hours	28.8 (15.3–113.8)	27.0 (13.9–115.1)	31.5 (15.4–113.8)	0.384
Treatment at AF onset				
Mechanical ventilation, n (%)	212 (68.8%)	148 (69.5%)	64 (67.4%)	0.711
Renal replacement therapy, n (%)	83 (27.0%)	67 (31.5%)	16 (16.8%)	0.008
Vasopressors, n (%)	157 (51.0%)	107 (50.2%)	50 (52.6%)	0.698
Inotropes, n (%)	39 (12.7%)	25 (11.7%)	14 (14.7%)	0.465

CHA2DS2-VASc score (Congestive heart failure; Hypertension; Age ≥ 75 years [2 points]; Diabetes; previous Stroke, transient ischemic attack, or thromboembolism [2 points]; Vascular disease; Age 65–74 years; and Female); range 0–9 points. A score ≥ 2 indicates “high risk.” HAS-BLED bleeding score (Hypertension, Abnormal renal/liver function, Stroke, Bleeding history or predisposition, Labile international normalized ratio, Elderly (> 65 years), Drugs/alcohol concomitantly); range 0–6 points. A score ≥ 3 indicates “high risk”

AF, atrial fibrillation; APACHE II, acute physiology and chronic health evaluation II; ICU, intensive care unit; SOFA, sequential organ failure assessment

Interventions and outcomes are shown in Table 2. The proportions of patients administered rhythm-and-rate-control drugs within the initial AF duration did not differ between the two groups. However, patients in the Early group had more frequent AF at ICU discharge. Direct-current cardioversion was performed less often in the Early group, albeit statistical significance. Most patients received continuous intravenous heparin injection as an initial anticoagulant. The Early group had a longer duration of anticoagulation therapy and fewer patients with first restoration of SR before anticoagulation therapy than the Non-early group. The incidence of the composite outcome tended to occur less frequently (Non-early 34.3% vs. Early 24.2%, *P* = 0.078) and significantly later in the Early group (*P* = 0.02 by log-rank test, Additional file 1: Figure S1). Bleeding complications occurred in 27 patients (8.8%), which were not significantly different between the two groups. Of these, 19 events (70.4%) were actionable (BARC types 2‒5). ICU and hospital length of stay were not different between the two groups.

**Table. 2:** Interventions and outcomes: non-early group vs. early group

	Overall N = 308	Non-early group N = 213	Early group N = 95	*P* value
AF duration, hours	18.1 (4.2–50.8)	16.2 (4.3–44.7)	20.4 (4–64.4)	0.392
AF duration ≥ 48 h, n (%)	81 (26.3%)	51 (23.9%)	30 (31.6%)	0.160
SR restoration before anticoagulation therapy^a^, n (%)	24 (18.8%)	16 (48.5%)	8 (8.4%)	< 0.01
AF recurrence, n (%)	83 (27.0%)	64 (30.1%)	19 (20.0%)	0.066
AF at ICU discharge^b^, n (%)	43 (16.0%)	23 (12.8%)	20 (22.7%)	0.037
Rhythm control drug, n (%)	120 (39.0%)	84 (39.4%)	36 (37.9%)	0.800
Rate control drug, n (%)	216 (70.1%)	150 (70.4%)	66 (69.5%)	0.867
DC, n (%)	56 (18.2%)	44 (20.7%)	12 (12.6%)	0.092
Initial anticoagulant^a^				0.340
Subcutaneous heparin injection	31 (24.2%)	7 (21.2%)	24 (25.3%)	
Continuous heparin intravenous injection	87 (68.0%)	21 (63.6%)	66 (69.5%)	
Warfarin	2 (1.6%)	1 (3.0%)	1 (1.1%)	
Direct oral anticoagulant	8 (6.3%)	4 (12.1%)	4 (4.2%)	
Anticoagulants during observation period in ICU				
Anticoagulants from AF onset, h^a^	11.4 (0–50.8)	82.7 (58.3–102.5)	1.5 (0–20.6)	< 0.01
Duration of anticoagulation therapy, h^a^	94.2 (44.3–160.6)	68.2 (30.4–92.4)	121.5 (65.4–167.6)	< 0.01
Anticoagulants at the end of observation period, n (%)	77 (25.0%)	24 (11.3%)	53 (55.8%)	0.082
Mortality or Ischemic stroke during hospital stay, n (%)	96 (31.2%)	73 (34.3%)	23 (24.2%)	0.078
ICU mortality, n (%)	40 (13.0%)	33 (15.5%)	7 (7.4%)	0.050
30-day mortality, n (%)	65 (21.1%)	52 (24.4%)	13 (13.7%)	0.063
Hospital mortality, n (%)	87 (28.3%)	65 (30.5%)	22 (23.2%)	0.185
Ischemic stroke until hospital discharge, n (%)	11 (3.6%)	10 (4.7%)	3 (3.1%)	0.112
During ICU stay n (%)	6 (2.0%)	5 (2.4%)	1 (1.1%)	0.448
After ICU discharge^b^, n (%)	5 (1.9%)	5 (2.8%)	0 (0%)	0.132
Bleeding complication^c^, n (%)	27 (8.8%)	22 (10.3%)	7 (7.4%)	0.518
Type 1	8	7	1	
Type 2	7	6	1	
Type 3	10	8	2	
Type 4	0	0	0	
Type 5	2	1	1	
ICU length of stay, days	8 (5–14)	8 (6–14)	8 (5–12)	0.270
Hospital length of stay, days	32 (17–56)	30 (16–59)	33 (19–53)	0.441

Rhythm control drugs used during AF from the initial onset are the following: amiodarone, pilsicainide, magnesium sulfate, and other any antiarrhythmic agents. Rate control drugs used during AF from the initial onset are the following: diltiazem, verapamil, landiolol, propranolol, other β-blocking agents, and digoxin. AF duration was calculated by the data of first AF event. Data were missing for BMI (1 patient) and SOFA score (3 patients)

AF, atrial fibrillation; DC, direct current cardioversion; ICU, intensive care unit; SR, sinus rhythm

^a^Excluded the patients who were not used any anticoagulants in ICU (n = 180)

^b^Excluded the patients who died in ICU (n = 40)

^c^Classified by Bleeding Academic Research Consortium (BARC) Definition for Bleeding

The results of the Cox models for the composite outcome after adjustment for the prespecified confounding factors are shown in Table 3. Among all covariates included in this model, early anticoagulation therapy did not improve the composite outcome (adjusted hazard ratio [HR] 0.77; 95% confidence interval [CI] 0.47‒1.23).

**Table. 3:** Estimates of the effect of covariates on hospital mortality or cerebral infarction in the Cox proportional hazards models

	Adjusted hazard ratio (95% CI)	*P* value
Anticoagulation therapy within 48 h from AF onset	0.77 (0.47–1.23)	0.281
Age (per 5 year old)	1.02 (0.92–1.15)	0.664
Male	1.11 (0.68–1.87)	0.680
APACHE II score (per 5 points)	1.05 (0.91–1.21)	0.490
Infection at AF onset	1.02 (0.60–1.82)	0.950
CHA2DS2-VASc score	1.04 (0.88–1.28)	0.743
HAS-BLED bleeding score	1.13 (0.86–1.47)	0.394
Mechanical ventilation at AF onset	1.24 (0.75–2.13)	0.418
Renal replacement therapy at AF onset	1.98 (1.20–3.27)	0.007

Adjusted by the following factors; age, sex, APACHE II score, CHA2DS2-VASc score, HAS-BLED bleeding score, Mechanical ventilation, Renal replacement therapy, and infection at AF onset

AF, atrial fibrillation; APACHE II, acute physiology and chronic health evaluation II

**Fig. 3:**
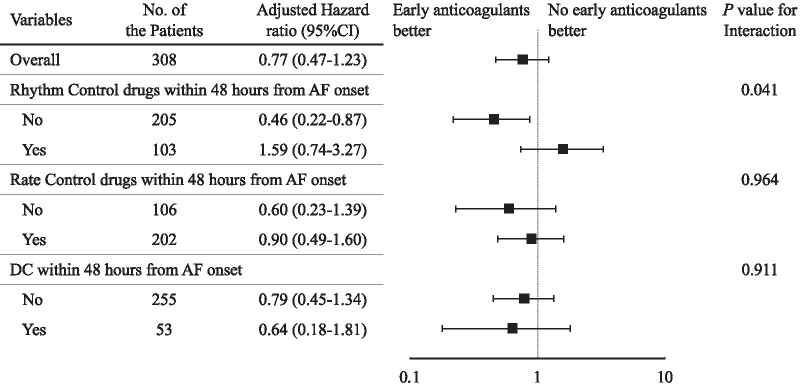
Primary outcome in the stratified groups. The primary outcome, which was the composite of mortality or cerebral infarction from AF onset to hospital discharge, was adjusted by the following factors: age, sex, APACHE II score, CHA2DS2-VASc score, HAS-BLED bleeding score, mechanical ventilation, renal replacement therapy, and infection at AF onset. Rhythm control drugs used during AF from the initial onset included amiodarone, pilsicainide, magnesium sulfate, and other any antiarrhythmic agents. Rate control drugs used during AF from the initial onset included diltiazem, verapamil, landiolol, propranolol, other β-blocking agents, and digoxin. AF, atrial fibrillation; DC, direct current cardioversion

The results of the Cox models in the stratified groups are shown in Fig. 3. The composite outcome occurred less frequently in Early group patients without rhythm control drugs (adjusted HR 0.46; 95% CI 0.22‒0.87). There was also a significant interaction between early anticoagulation therapy and rhythm control drugs.

## Discussion

### Key findings

This post-hoc analysis of a prospective multicenter observational study assessed the efficacy of early anticoagulation therapy for critically ill patients with NOAF. Among the 308 patients studied, 128 (41.6%) received anticoagulation therapy during the observation period. Of these, anticoagulation therapy was initiated within 48 h after AF onset in 95 patients (74.2%). After dividing the patients into four groups based on CHA2DS2-VASc and HAS-BLED bleeding scores, we found that the proportion of anticoagulation therapy administered was not different among the four groups. More than half of the patients had a high risk of stroke (CHA2DS2-VASc score ≥ 2) and low risk of bleeding (HAS-BLED bleeding score < 3); but however, less than half of these patients received anticoagulation therapy. Patients in the Early group, who had lower severity scores and were less frequently treated with RRT at AF onset, tended to have fewer composite outcomes than those in the Non-early group. However, after adjustment using a multivariable Cox regression model, we noted that early anticoagulation therapy did not decrease the composite outcome. A significant interaction term was identified between early anticoagulation therapy and rhythm control drugs, and the composite outcome occurred less frequently in Early group patients who did not receive rhythm control drugs. The incidence of bleeding complications was not different between the two groups.

### Comparison with previous studies

Previous observational studies evaluating anticoagulation therapy in critically ill patients with AF have reported that anticoagulants were prescribed in only 3.1‒37.6% patients and that the incidence of ischemic stroke and bleeding complications was 0‒2.7% and 7.6‒8.6% during hospital stay, respectively [18–21, 25]. The use of anticoagulation therapy in critically ill patients is challenging because the incidence of bleeding events outweighs that of thromboembolic events, unlike in a general hospital setting, in which the annual incidence of ischemic stroke and bleeding complications is similar [12, 15]. We found similar patient characteristics, including age and CHA2DS2-VASc score, and comparable incidence of ischemic stroke (3.6%) and bleeding complications (8.8%) to those in previous studies mentioned above. In our study, 41.6% of patients were administered with anticoagulants. Our findings imply that anticoagulation therapy is not commonly administered in critically ill patients with NOAF.

Although 82.2% of the critically ill patients with NOAF had a CHA2DS2-VASc score of ≥ 2 [22], this score did not consistently predict the risk of ischemic stroke and mortality in critically ill patients during hospitalization [21, 33, 34]. To date, bleeding scores had not been evaluated in critically ill patients. In our study, 79.2% of the included patients had a CHA2DS2-VASc score ≥ 2, similar to a previous study [22], and 26.6% of the included patients were identified to have a high risk of bleeding based on the HAS-BLED bleeding risk score. Less than half of the patients who had indications for anticoagulation therapy, as evaluated by these scores, received anticoagulation therapy. The proportion of patients receiving anticoagulation therapy was similar among the groups stratified by these two scores. The use of these scores to decide on anticoagulation therapy implementation may not yet be widely accepted in critically ill patients with NOAF. Further trials with long follow-up periods are needed to evaluate these scores in critically ill patients with NOAF. After resolving critical illness, however, anticoagulants may need to be administered for at least 30 days to patients with high risk of stroke based on the clinical prediction scores even after SR restoration [14].

Renal dysfunction could contribute to an increased risk of stroke via procoagulant and inflammatory pathways and changes in arterial compliance/stiffness [35]. In addition to the CHA2DS2-VASc score, chronic kidney disease and persistent AF were identified as independent risk factors for left atrial appendage thrombus in the general population [36]. In our study, the proportions of patients with chronic hemodialysis and RRT at AF onset were 5.5% and 27.0%, respectively. Using a multivariable Cox proportional hazard model, RRT was found to be a significant risk factor for poor neurological outcome. This finding implies that not only chronic kidney disease but also acute kidney injury may be associated with poor outcomes, similar to that in the general population.

Only a few studies have evaluated the relationship between anticoagulation therapy and mortality in critically ill patients with NOAF. In a retrospective observational study, anticoagulation therapy decreased hospital mortality, but without statistical significance (25.7% vs. 33.8%, *P* = 0.392), in septic patients with AF. Although ischemic stroke was not noted observed in this study, bleeding complications occurred only in the patients who received anticoagulation therapy (0% vs. 5.7%, *P* = 0.031) [25]. In 2016, Walkey et al. [21] studied the effect of anticoagulation therapy among 27,010 septic patients using propensity score matching. They found that parenteral anticoagulants did not decrease the incidence of in-hospital ischemic stroke (relative risk [RR], 0.94; 95% CI, 0.77‒1.15), despite a significant increase in bleeding (RR, 1.21; 95% CI, 1.10‒1.32). In our study, the incidence of bleeding complications was not increased, but patients in the Early group had lower severity scores. This might imply that anticoagulants were administered in patients whom clinicians considered as having a low risk of bleeding complications. Although mortality and ischemic stroke tended to be less common in the Early group, after adjustment using a multivariable Cox proportional hazard model, early anticoagulation therapy did not improve clinical outcomes. We needed to use a composite outcome for multivariable adjustment because of the small sample size; hence, further studies with a larger cohort are required to evaluate the impact of early anticoagulation therapy on mortality and ischemic stroke.

### Significance and implications

Our findings imply that the clinical scores for predicting the risk of ischemic stroke or bleeding complications may not commonly be used to decide the implementation of anticoagulation therapy and that routine early anticoagulation therapy may not be effective in critically ill patients with NOAF. Because anticoagulation should be performed only in patients with higher stroke risk than bleeding risk, clinical scores validated in critically ill patients with AF are needed.

The AFTER-ICU study showed that longer AF duration contributed to higher hospital mortality [24]. In a post-hoc analysis of the AFTER-ICU study, using a multivariable Cox proportional hazard model, the use of rhythm control drugs was associated with SR restoration (adjusted HR, 1.46; 95% CI, 1.16‒1.85) and decreased AF at ICU discharge (10.1% vs. 21.4%, *P* = 0.004) [37]. Restoration of SR in patients with AF is considered a logical strategy to improve clinical outcomes by preventing thromboembolic complications [38]. We identified an interaction term between early anticoagulation therapy and rhythm control drugs and found that the composite outcome was less frequent in Early group patients not receiving rhythm control drugs. The use of rhythm control drugs may reduce the benefit of anticoagulation therapy. Further investigation considering this interaction is needed.

### Strengths and limitations

To the best of our knowledge, this is the first study to describe the epidemiology of anticoagulation therapy for critically ill patients with NOAF according to the CHA2DS2-VASc and HAS-BLED scores and assess the efficacy of early anticoagulation therapy in these patients.

However, this study also had several limitations. First, because of the observational nature of the study, we could not control for unmeasured or unknown confounding factors that may have influenced the results. For example, we did not collect information on antiplatelet therapy, blood transfusion, and the management of underlying diseases in critically ill patients. Second, it is unclear whether anticoagulants were administered effectively to prevent ischemic stroke. For example, laboratory data were not collected to evaluate whether the anticoagulation therapy was in the therapeutic range. More than half of the anticoagulants were infused continuously, and oral anticoagulants were rarely used, possibly because of concerns on poor enteral absorption in critically ill patients [39, 40]. The administration of anticoagulants that are within appropriate therapeutic ranges may be needed for patients with higher risks of stroke. Approximately half of the anticoagulants were discontinued during the observational period; this duration was shorter than the recommended guideline of continuing anticoagulants for at least 30 days without any interruptions [14]. Thus, further studies are needed to evaluate standardized anticoagulation therapy with appropriate monitoring and duration. Third, more than half of the patients in our study were medical patients. Early anticoagulation therapy would not be acceptable in surgical patients because of the higher risk of bleeding [41, 42]. Low-molecular weight heparin was never used because of the lack of indication for non-surgical patients in Japan. Fourth, although we attempted to detect all NOAF events including asymptomatic ones, it is likely that we did not detect some NOAF events that could not be clinically recognized. However, a retrospective cohort study using an automated analysis of continuous electrocardiogram of critically ill patients found that subclinical AF might not be associated with poor hospital outcomes [6]. Finally, because the study was conducted only in Japan, our findings may have limited generalizability for other countries. However, the incidences of ischemic stroke, bleeding complications, and hospital mortality were similar to those reported in previous studies [9, 10, 21].

## Conclusion

In this study, we found that less than half of the critically ill patients with NOAF received anticoagulation therapy. We also found that clinical prediction scores were not used to decide anticoagulation therapy implementation and that early anticoagulation therapy did not improve clinical outcomes in critically ill patients with NOAF. Further studies are needed to evaluate the efficacy of anticoagulation therapy in critically ill patients with NOAF, preferably considering the interaction of rhythm control strategy.

## Supplementary Information

**Additional file 1.** Supplementary information on the methods and further results

## Data Availability

The datasets used and/or analyzed during the current study are available from the corresponding author on reasonable request.

## Abbreviations

AF: Atrial fibrillation
APACHE II: Acute Physiology and Chronic Health Evaluation II
BARC: Bleeding Academic Research Consortium
CI: Confidence interval
DC: Direct-current cardioversion
HR: Hazard ratio
ICU: Intensive care unit
NOAF: New-onset atrial fibrillation
RRT: Renal replacement therapy
SOFA: Sequential Organ Failure Assessment
SR: Sinus rhythm

## References

[1] Annane D, Sebille V, Duboc D (2008). Incidence and prognosis of sustained arrhythmias in critically ill patients. Am J Respir Crit Care Med.

[2] Bender JS (1996). Supraventricular tachyarrhythmias in the surgical intensive care unit: an under-recognized event. Am Surg.

[3] Knotzer H, Mayr A, Ulmer H (2000). Tachyarrhythmias in a surgical intensive care unit: a case-controlled epidemiologic study. Intensive Care Med.

[4] Alonso-Coello P, Cook D, Xu SC (2017). Predictors, prognosis, and management of new clinically important atrial fibrillation after noncardiac surgery: a prospective cohort study. Anesth Analg.

[5] Gialdini G, Nearing K, Bhave PD (2014). Perioperative atrial fibrillation and the long-term risk of ischemic stroke. JAMA.

[6] Moss TJ, Calland JF, Enfield KB (2017). New-onset atrial fibrillation in the critically ill. Crit Care Med.

[7] Sibley S, Muscedere J (2015). New-onset atrial fibrillation in critically ill patients. Can Respir J.

[8] Walkey AJ, Hammill BG, Curtis LH (2014). Long-term outcomes following development of new-onset atrial fibrillation during sepsis. Chest.

[9] Yoshida T, Fujii T, Uchino S (2015). Epidemiology, prevention, and treatment of new-onset atrial fibrillation in critically ill: a systematic review. J Intensive Care.

[10] Kuipers S, Klein Klouwenberg PM, Cremer OL (2014). Incidence, risk factors and outcomes of new-onset atrial fibrillation in patients with sepsis: a systematic review. Crit Care.

[11] Gage BF, Waterman AD, Shannon W (2001). Validation of clinical classification schemes for predicting stroke: results from the National Registry of Atrial Fibrillation. JAMA.

[12] Lip GY, Nieuwlaat R, Pisters R (2010). Refining clinical risk stratification for predicting stroke and thromboembolism in atrial fibrillation using a novel risk factor-based approach: the euro heart survey on atrial fibrillation. Chest.

[13] Lip GY, Frison L, Halperin JL (2010). Identifying patients at high risk for stroke despite anticoagulation: a comparison of contemporary stroke risk stratification schemes in an anticoagulated atrial fibrillation cohort. Stroke.

[14] January CT, Wann LS, Alpert JS (2014). 2014 AHA/ACC/HRS guideline for the management of patients with atrial fibrillation: executive summary: a report of the American College of Cardiology/American Heart Association Task Force on practice guidelines and the Heart Rhythm Society. Circulation.

[15] Pisters R, Lane DA, Nieuwlaat R (2010). A novel user-friendly score (HAS-BLED) to assess 1-year risk of major bleeding in patients with atrial fibrillation: the Euro Heart Survey. Chest.

[16] Gallego P, Roldan V, Torregrosa JM (2012). Relation of the HAS-BLED bleeding risk score to major bleeding, cardiovascular events, and mortality in anticoagulated patients with atrial fibrillation. Circ Arrhythm Electrophysiol.

[17] Garcia-Fernandez A, Marin F, Roldan V (2016). The HAS-BLED score predicts long-term major bleeding and death in anticoagulated non-valvular atrial fibrillation patients undergoing electrical cardioversion. Int J Cardiol.

[18] Koyfman L, Brotfain E, Kutz R (2015). Epidemiology of new-onset paroxysmal atrial fibrillation in the General Intensive Care Unit population and after discharge from ICU. A retrospective epidemiological study. Anaesthesiol Intensive Ther.

[19] Salman S, Bajwa A, Gajic O (2008). Paroxysmal atrial fibrillation in critically ill patients with sepsis. J Intensive Care Med.

[20] Kanji S, Williamson DR, Yaghchi BM (2012). Epidemiology and management of atrial fibrillation in medical and noncardiac surgical adult intensive care unit patients. J Crit Care.

[21] Walkey AJ, Quinn EK, Winter MR (2016). Practice patterns and outcomes associated with use of anticoagulation among patients with atrial fibrillation during sepsis. JAMA Cardiol.

[22] Schoaps RS, Quintili A, Bonavia A (2019). Stroke prophylaxis in critically-ill patients with new-onset atrial fibrillation. J Thromb Thrombolysis.

[23] Garg A, Khunger M, Seicean S (2016). Incidence of thromboembolic complications within 30 days of electrical cardioversion performed within 48 hours of atrial fibrillation onset. JACC Clin Electrophysiol.

[24] Yoshida T, Uchino S, Sasabuchi Y (2020). Prognostic impact of sustained new-onset atrial fibrillation in critically ill patients. Intensive Care Med.

[25] Darwish OS, Strube S, Nguyen HM (2013). Challenges of anticoagulation for atrial fibrillation in patients with severe sepsis. Ann Pharmacother.

[26] Knaus WA, Draper EA, Wagner DP (1985). APACHE II: a severity of disease classification system. Crit Care Med.

[27] Vincent JL, Moreno R, Takala J (1996). The SOFA (Sepsis-related Organ Failure Assessment) score to describe organ dysfunction/failure. On behalf of the Working Group on Sepsis-Related Problems of the European Society of Intensive Care Medicine. Intensive Care Med.

[28] Mitric G, Udy A, Bandeshe H (2016). Variable use of amiodarone is associated with a greater risk of recurrence of atrial fibrillation in the critically ill. Crit Care.

[29] Mehran R, Rao SV, Bhatt DL (2011). Standardized bleeding definitions for cardiovascular clinical trials: a consensus report from the Bleeding Academic Research Consortium. Circulation.

[30] Czempik P, Cieśla D, Knapik P (2018). Mortality of patients with acute kidney injury requiring renal replacement therapy. Adv Clin Exp Med.

[31] Colpan A, Akinci E, Erbay A (2005). Evaluation of risk factors for mortality in intensive care units: a prospective study from a referral hospital in Turkey. Am J Infect Control.

[32] Song JE, Kim MH, Jeong WY (2016). Mortality risk factors for patients with septic shock after implementation of the surviving sepsis campaign bundles. Infect Chemother.

[33] Karamchandani K, Schoaps RS, Abendroth T (2020). CHA2DS2-VASc score and in-hospital mortality in critically ill patients with new-onset atrial fibrillation. J Cardiothorac Vasc Anesth.

[34] Champion S, Lefort Y, Gauzere BA (2014). CHADS2 and CHA2DS2-VASc scores can predict thromboembolic events after supraventricular arrhythmia in the critically ill patients. J Crit Care.

[35] Floria M, Tanase DM (2019). Atrial fibrillation type and renal dysfunction: new challenges in thromboembolic risk assessment. Heart.

[36] Kapłon-Cieślicka A, Budnik M, Gawałko M (2019). Atrial fibrillation type and renal dysfunction as important predictors of left atrial thrombus. Heart.

[37] Yoshida T, Uchino S, Sasabuchi Y (2021). Rhythm-control therapy for new-onset atrial fibrillation in critically ill patients: a post hoc analysis from the prospective multicenter observational AFTER-ICU study. IJC Heart Vasc.

[38] Sherman DG (2007). Stroke prevention in atrial fibrillation: pharmacological rate versus rhythm control. Stroke.

[39] Peterson JJ, Hoehns JD (2016). Administration of direct oral anticoagulants through enteral feeding tubes. J Pharm Technol.

[40] Smith BS, Yogaratnam D, Levasseur-Franklin KE (2012). Introduction to drug pharmacokinetics in the critically ill patient. Chest.

[41] Malouf JF, Alam S, Gharzeddine W (1993). The role of anticoagulation in the development of pericardial effusion and late tamponade after cardiac surgery. Eur Heart J.

[42] Dunning J, Nagarajan DV, Amanullah M (2004). What is the optimal anticoagulation management of patients post-cardiac surgery who go into atrial fibrillation?. Interact Cardiovasc Thorac Surg.

