# Supplementary material for: Clinical prediction scores and early anticoagulation therapy for new-onset atrial fibrillation in critical illness: a post-hoc analysis
Source: BMC Cardiovasc Disord. 2021 Sep 8;21:423. doi: 10.1186/s12872-021-02235-8 (PMC8424957; doi:10.1186/s12872-021-02235-8)
Supplement: Supplementary file 1 — Additional file 1. Supplementary information on the methods and further results [file 12872_2021_2235_MOESM1_ESM.docx]

**Clinical prediction scores and early anticoagulation therapy for new-onset atrial fibrillation in critical illness: A post-hoc analysis**

Supplementary information on the methods and further results

Table of Contents

*Supplemental tables*

Table S1. Bleeding Academic Research Consortium Definition for Bleeding

...............................................................................................2

Table S2. Detail of CHA2DS2-VASc score

...............................................................................................4

Table S3. e-Table 3. Detail of HAS-BLED bleeding risk score

...............................................................................................5

Table S4. Physiological data and laboratory tests

...............................................................................................6

*Supplemental figure*

Figure S1. Kaplan-Meier estimate for survival without ischemic stroke

...............................................................................................8

**Table S1. Bleeding events as defined by the Bleeding Academic Research Consortium** [1]

| Type | | Bleeding definition |
| --- | --- | --- |
| Type 0 | | No bleeding |
| Type 1 | | Bleeding that is not actionable and does not cause the patient to seek unscheduled performance of studies, hospitalization, or treatment by a healthcare professional; may include episodes leading to self-discontinuation of medical therapy by the patient without consulting a healthcare professional |
| Type 2 | | Any overt, actionable sign of hemorrhage (eg, more bleeding than would be expected for a clinical circumstance, including bleeding found by imaging alone) that does not fit the criteria for type 3, 4, or 5 but does meet at least one of the following criteria: (1) requiring nonsurgical, medical intervention by a healthcare professional, (2) leading to hospitalization or increased level of care, or (3) prompting evaluation |
| Type 3 | 3a | Overt bleeding plus hemoglobin drop of 3 to < 5 g/dL ^a^ (provided hemoglobin drop is related to bleed) Any transfusion with overt bleeding |
|  | 3b | Overt bleeding plus hemoglobin drop ≥ 5 g/dL ^a^ (provided hemoglobin drop is related to bleed) Cardiac tamponade Bleeding requiring surgical intervention for control (excluding dental/nasal/skin/hemorrhoid) Bleeding requiring intravenous vasoactive agents |
|  | 3c | Intracranial hemorrhage (does not include microbleeds or hemorrhagic transformation, does include intraspinal) Subcategories confirmed by autopsy or imaging or lumbar puncture Intraocular bleed compromising vision |
| Type 4  CABG-related bleeding | | Perioperative intracranial bleeding within 48 h Reoperation after closure of sternotomy for the purpose of controlling bleeding Transfusion of ≥ 5 U whole blood or packed red blood cells within a 48-h period ^b^ Chest tube output ≥ 2L within a 24-h period |
| Type 5  Fatal bleeding | 5a | Probable fatal bleeding; no autopsy or imaging confirmation but clinically suspicious |
|  | 5b | Definite fatal bleeding; overt bleeding or autopsy or imaging confirmation |

CABG indicates coronary artery bypass graft. Platelet transfusions should be recorded and reported but are not included in these definitions until further information is obtained about the relationship to outcomes. If a CABG-related bleed is not adjudicated as at least a type 3 severity event, it will be classified as not a bleeding event. If a bleeding event occurs with a clear temporal relationship to CABG (ie, within a 48-h time frame) but does not meet type 4 severity criteria, it will be classified as not a bleeding event.

a. Corrected for transfusion (1 U packed red blood cells or 1 U whole blood = 1 g/dL hemoglobin).

b. Cell saver products are not counted.

1. Mehran R, Rao SV, Bhatt DL, Gibson CM, Caixeta A, Eikelboom J, Kaul S, Wiviott SD, Menon V, Nikolsky E et al: Standardized bleeding definitions for cardiovascular clinical trials: a consensus report from the Bleeding Academic Research Consortium. Circulation 2011, 123(23):2736-2747.

**Table S2. Detail of CHA2DS2-VASc score** [1]

|  | Overall  N=308 | Non-early group  N=213 | Early group  N=95 | *P* value |
| --- | --- | --- | --- | --- |
| Congestive Heart Failure, n (%) | 26 (8.4%) | 20 (9.4%) | 6 (6.3%) | 0.370 |
| Hypertension, n (%) | 177 (57.5%) | 121 (56.8%) | 56 (59.0%) | 0.726 |
| Age ≥ 75, n (%) | 156 (50.6%) | 106 (49.8%) | 50 (52.6%) | 0.642 |
| Diabetes mellitus, n (%) | 83 (27.0%) | 61 (28.6%) | 22 (23.2%) | 0.317 |
| Stroke/TIA, n (%) | 32 (10.4%) | 21 (9.9%) | 11 (11.6%) | 0.648 |
| Vascular disease, n (%) | 24 (7.8%) | 17 (8.0%) | 7 (7.4%) | 0.853 |
| Age 65-74, n (%) | 85 (27.6%) | 59 (27.7%) | 26 (27.4%) | 0.952 |
| Female, n (%) | 100 (32.5%) | 69 (32.4%) | 31 (32.6%) | 0.967 |

Hypertension was defined as systolic blood pressure >140 mmHg without any vasopressors or history.

Age ≥ 75 and Stroke/TIA are counted as 2 points.

Other variables are counted as 1 point.

1. Lip GY, Nieuwlaat R, Pisters R, Lane DA, Crijns HJ: Refining clinical risk stratification for predicting stroke and thromboembolism in atrial fibrillation using a novel risk factor-based approach: the euro heart survey on atrial fibrillation. Chest 2010, 137(2):263-272.

Table S3. Detail of HAS-BLED bleeding risk score [1]

|  | Overall  N=308 | Non-early group  N=213 | Early group  N=95 | *P* value |
| --- | --- | --- | --- | --- |
| Hypertension, n (%) | 154 (50.0%) | 105 (49.3%) | 49 (51.6%) | 0.711 |
| Abnormal renal function, n (%) | 80 (30.0%) | 62 (29.1%) | 18 (19.0%) | 0.060 |
| Abnormal liver function, n (%) | 62 (20.1%) | 49 (23.0%) | 13 (13.7%) | 0.060 |
| Stroke, n (%) | 32 (10.4%) | 21 (9.9%) | 11 (11.6%) | 0.648 |
| Bleeding, n (%) | 1 (0.3%) | 0 (0%) | 1 (1.1%) | 0.214 |
| Labile INRs, n (%) | 7 (2.3%) | 4 (1.9%) | 3 (3.2%) | 0.486 |
| Elderly (≥65), n (%) | 241 (78.3%) | 165 (77.5%) | 76 (80.0%) | 0.618 |
| Drugs or Alcohol use, n (%) | 24 (7.8%) | 17 (8.0%) | 7 (7.4%) | 0.852 |

Hypertension was defined as systolic blood pressure >160 mmHg without any vasopressors or history.

Abnormal kidney function was defined as the presence of chronic dialysis or renal transplantation or serum creatinine ≥200 µmol/L.

Abnormal liver function was defined as chronic hepatic disease (e.g. cirrhosis) or biochemical evidence of significant hepatic derangement (e.g. bilirubin >2 x upper limit of normal).

Bleeding history was defined as any bleeding events before anticoagulants in ICU.

Alcohol use was not collected in the original study.

Each variable is counted as 1 point.

1. Pisters R, Lane DA, Nieuwlaat R, de Vos CB, Crijns HJ, Lip GY: A novel user-friendly score (HAS-BLED) to assess 1-year risk of major bleeding in patients with atrial fibrillation: the Euro Heart Survey. Chest 2010, 138(5):1093-1100.

Table S4. Physiological data and laboratory tests

|  | Overall  N=308 | Non-early group  N=213 | Early group  N=95 | *P* value |
| --- | --- | --- | --- | --- |
| Physiological data before AF onset |  |  |  |  |
| Heart rate, bpm | 96 (84-109) | 97 (83-110) | 93 (84-105) | 0.179 |
| Mean arterial pressure, mmHg | 80 (70-92) | 81 (71-92) | 77 (69-91) | 0.185 |
| Physiological data and laboratory tests at AF onset | |  |  |  |
| Heart rate, bpm | 131 (113-150) | 133 (111-151) | 129 (115-148) | 0.982 |
| Mean arterial pressure, mmHg | 76 (64-88) | 76 (64-86) | 72 (65-88) | 0.489 |
| F_I_O_2_ | 0.4 (0.3-0.44) | 0.4 (0.3-0.45) | 0.36 (0.3-0.4) | 0.782 |
| pH | 7.398 (7.345-7.450) | 7.392 (7.343-7.440) | 7.418 (7.348-7.460) | 0.063 |
| pCO_2_, Torr | 39.0 (34.4-44.9) | 38.9 (34.4-44.9) | 39.8 (33.9-45.2) | 0.831 |
| pO_2_, Torr | 89.6 (76.0-111.0) | 89.5 (77.3-112.4) | 89.8 (72.4-109.6) | 0.238 |
| Lactate, mol/L | 1.6 (1.19-2.5) | 1.7 (1.2-2.7) | 1.5 (1.1-2.0) | 0.042 |
| White blood cell count, ×10^3^/L | 10.3 (6.2-15.7) | 9.7 (5.6-15.2) | 10.6 (7.7-16.3) | 0.131 |
| Platelet count, ×10^3^/L | 104.5 (51.3-182.8) | 89 ( 44.5-171) | 135 (72-202) | 0.002 |
| PT-INR | 1.26 (1.11-1.43) | 1.26 (1.11-1.46) | 1.22 (1.1-1.40) | 0.110 |
| aPTT, seconds | 42.7 (33.6-54.3) | 43.1 (33.7-55.7) | 41.9 (33.4-49.4) | 0.179 |
| Bilirubin, mg/dL | 1 (0.6-2.0) | 1.2 (0.6-2.3) | 0.8 (0.5-1.5) | 0.006 |
| Creatinine, mg/dL | 1.27 (0.8-2.3) | 1.34 (0-.86-2.64) | 1.16 (0.66-1.96) | 0.023 |
| Blood urea nitrogen, mg/dL | 34.4 (21.0-50.0) | 34.8 (21.0-51.5) | 32.4 (21.0-48.0) | 0.609 |
| Sodium, mmol/L | 140 (137-143) | 139 (137-143) | 141 (137-144) | 0.066 |
| Potassium, mmol /L | 4.0 (3.7-4.5) | 4.1 (3.7-4.5) | 4.0 (3.7-4.6) | 0.991 |
| Chloride, mmol/L | 107 (103-110) | 106 (103-109) | 108 (104-111) | 0.037 |
| Magnesium, mmol/L | 2 (1.8-2.4) | 2 (1.8-2.4) | 2 (1.8-2.5) | 0.953 |
| CRP, mg/dL | 13.2 (5.6-23.8) | 12.8 (5.90-22.5) | 16.3 (4.9-25.4) | 0.304 |

Data were missing for PT-INR (21 patients), aPTT (23 patients), serum creatinine (1 patient), total bilirubin (2 patients).

Abbreviation: AF, atrial fibrillation; aPTT, activated partial thromboplastin time; CRP, C-reactive protein; PT-INR, prothrombin time - international normalized ratio.

Figure S1. Kaplan-Meier estimate for survival without ischemic stroke
